# Supplementary material for: Normoglycemia and physiological cortisone level maintain glucose homeostasis in a pancreas-liver microphysiological system
Source: Commun Biol. 2024 Jul 18;7:877. doi: 10.1038/s42003-024-06514-w (PMC11258270; doi:10.1038/s42003-024-06514-w)
Supplement: Supplementary file 2 — Supplementary Information [file 42003_2024_6514_MOESM2_ESM.pdf]

Supplementary Materials for

Normoglycemia and physiological cortisone level maintain glucose homeostasis in a pancreas-liver microphysiological system

Sophie Rigal *et al.*

Corresponding author. Email: liisa.vilen@astrazeneca.com

This PDF file includes:

Figs. S1 to S12
Tables S1 to S3

Fig. S1.

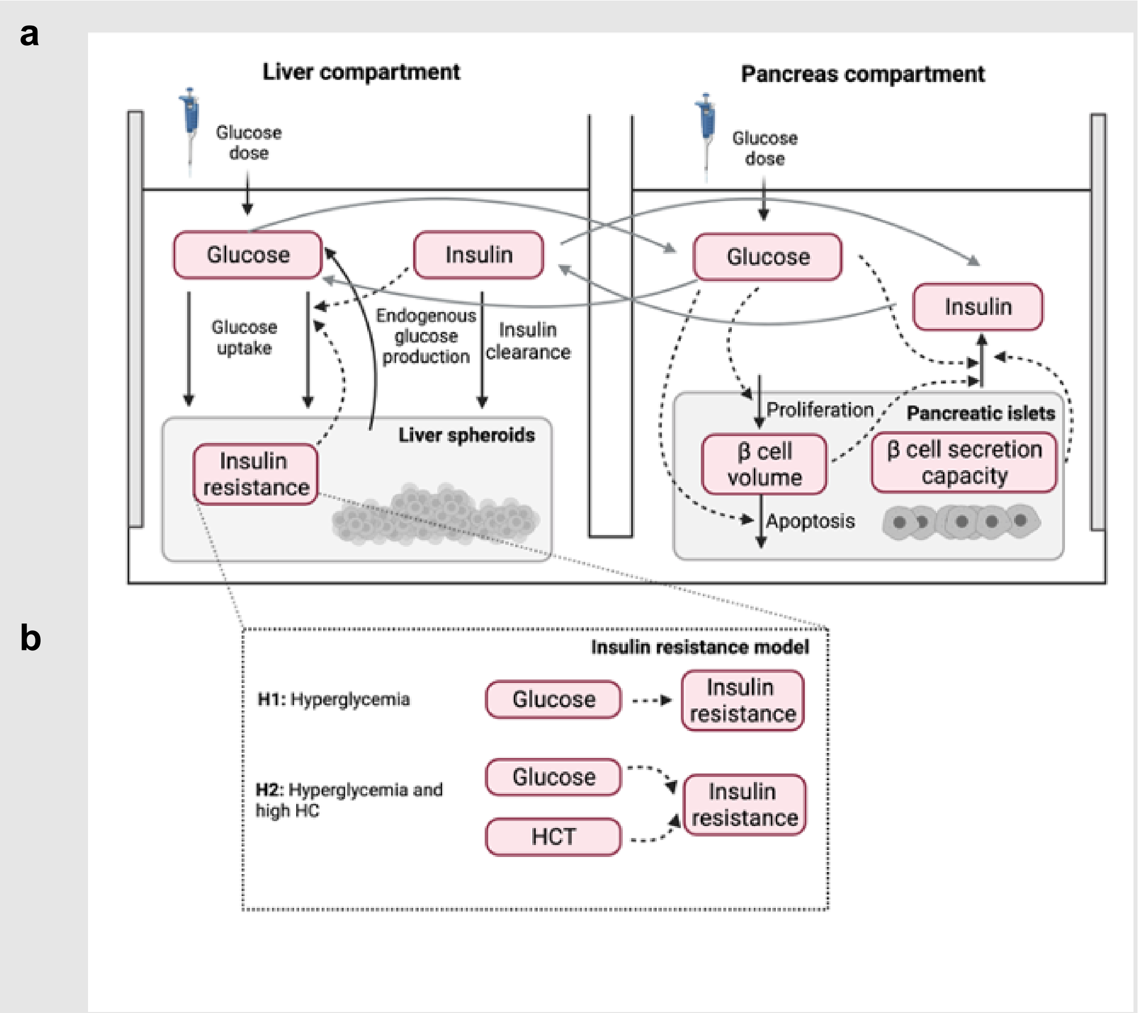

Overview of the mathematical model to study the development of insulin resistance in the pancreas-liver co-culture. (a) Previously reported mathematical model describing glucose metabolism in the pancreas-liver co-culture[14]. The model describes the behavior of key physiological variables involved in glucose homeostasis (red text boxes) and includes both liver (left) and pancreas (right) compartments, which represent the corresponding co-culture compartments in the MPS. The dashed arrows represent the interactions between the physiological variables described in the model, and the solid arrows represent metabolic fluxes in the co-culture. **(b)** Implementation of the hypotheses to study the development of insulin resistance. In the first hypothesis (H1), the insulin resistance variable is determined by the glucose content in the co-culture medium. In the second hypothesis (H2), insulin resistance is dependent on both glucose content and the concentration of hydrocortisone (HCT).

Fig. S2.

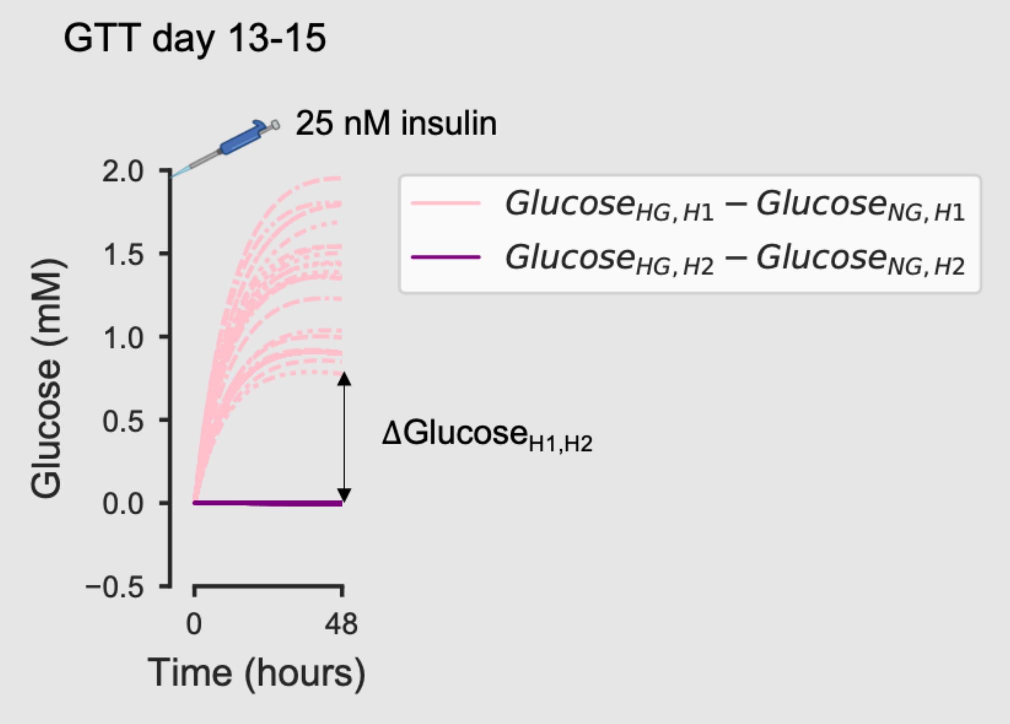

***In silico*-based calculation of the insulin dose to spike into the co-culture for differentiating between hypotheses.** We used the mathematical model calibrated using data from GTTs on days 1-3 and 7-9 to predict the effect of an insulin dose on glucose regulation for the studied hypothesis H1 and H2 (Fig. 2b). The lines represent model-predicted differences in glucose concentration between hyper- and normoglycemic co-cultures during the GTT on days 13-15 for both hypotheses H1 (pink lines) and H2 (purple lines) when a certain insulin dose is spiked into the co-culture medium. We simulated these glucose concentrations for a range of insulin doses using the mathematical model and selected the dose that yielded a concentration difference between the hypotheses ($G_{H1,H2}$) larger than the average SEM across all glucose experimental measurements on days 1-3 and days 7-9 (0.48 mM). The simulations shown in the figure correspond to the selected insulin dose (25 nM), where each line represents a model prediction with an acceptable parameter set.

Fig. S3.

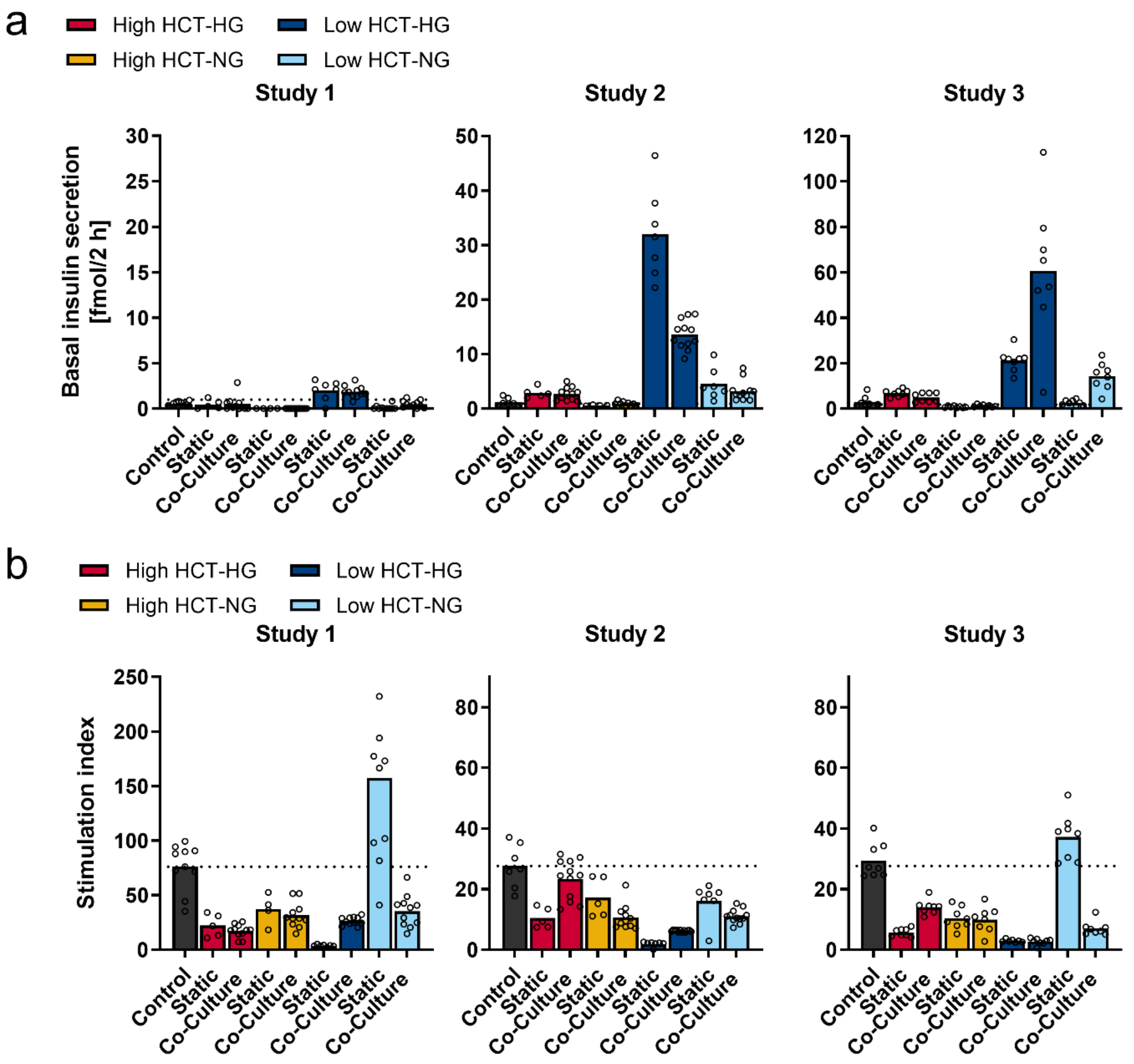

Effect of hydrocortisone and glucose level on islet functionality. (**a**) Basal insulin secretion in a low glucose solution (2.8 mM glucose) and **(b)** stimulation index showing the fold change in insulin secretion between low and high glucose solutions. Islets were cultured for 15 days in static mono-culture or in chip-based co-culture in the four different co-culture media. Islets cultured for 15 days in static monoculture in culture medium provided by the manufacturer served as a control (black bar and dotted line). Islets were extracted from the MPS to perform the GSIS assay with individual islets. Bars show mean and symbols represent individual islets (n values summarized in Table S1). An individual donor was used for each study (Table S2). Studies 1 and 2 were performed at TissUse and study 3 at AstraZeneca.

Fig. S4.

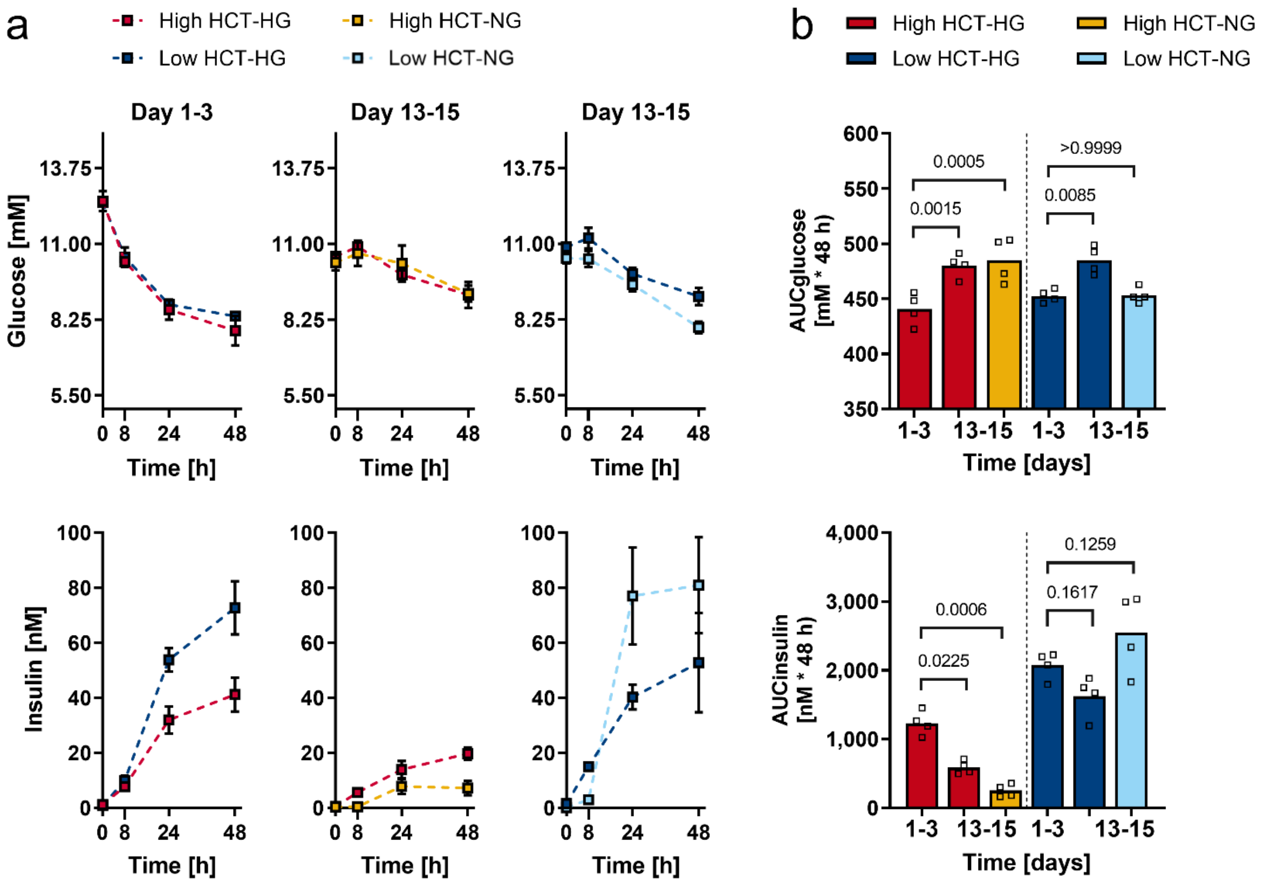

Physiological cortisone level maintains glucose tolerance in the pancreas-liver MPS. (**a**) Glucose and insulin concentration curves during glucose tolerance test. Data shown as mean ± SD (n = 4 individual co-culture replicates). (**b**) Area under the curve (AUC) for glucose (left) and insulin (right). Bars show mean and symbols represent individual co-culture replicates (circuits) from study 2 performed at TissUse (n = 4). Differences between selected pairs of conditions (day 13-15 compared to day 1-3) were evaluated by one-way ANOVA using Sidak’s multiple comparisons post-hoc test.

Fig. S5.

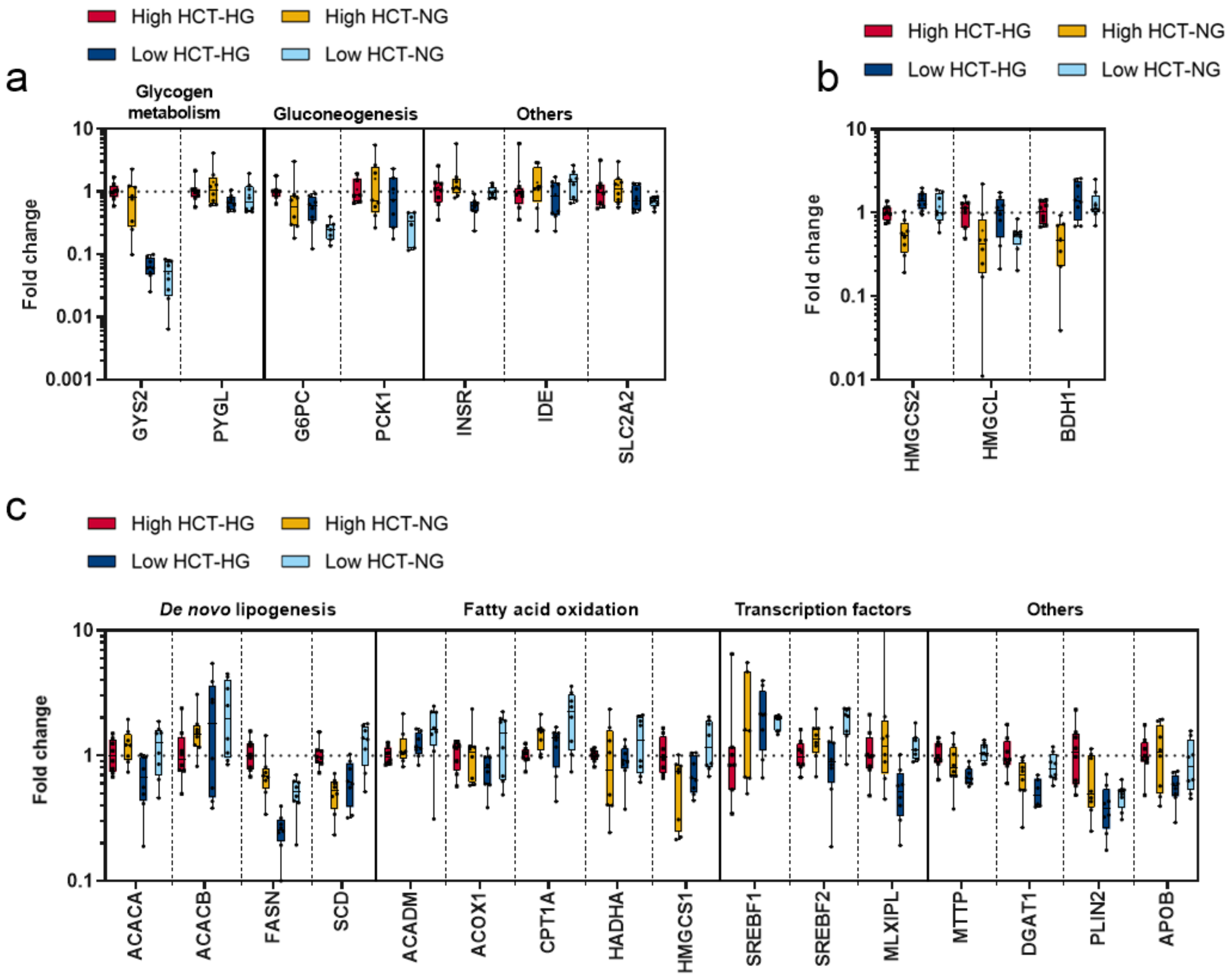

Effect of hydrocortisone and glucose level on gene expression in HepaRG/HHSteC liver spheroids. (a, b, c) Gene expression of enzymes involved in hepatic glucose metabolism (**a**) ketone body synthesis (**b**) and lipid metabolism (**c**). Data shown as fold change to High HCT-HG condition in a box-whisker plot with median and min-max values. Symbols represent liver samples from individual co-culture replicates from study 1 and 2 performed at TissUse (Study 1: n = 4, Study 2: n = 4).

Fig. S6.

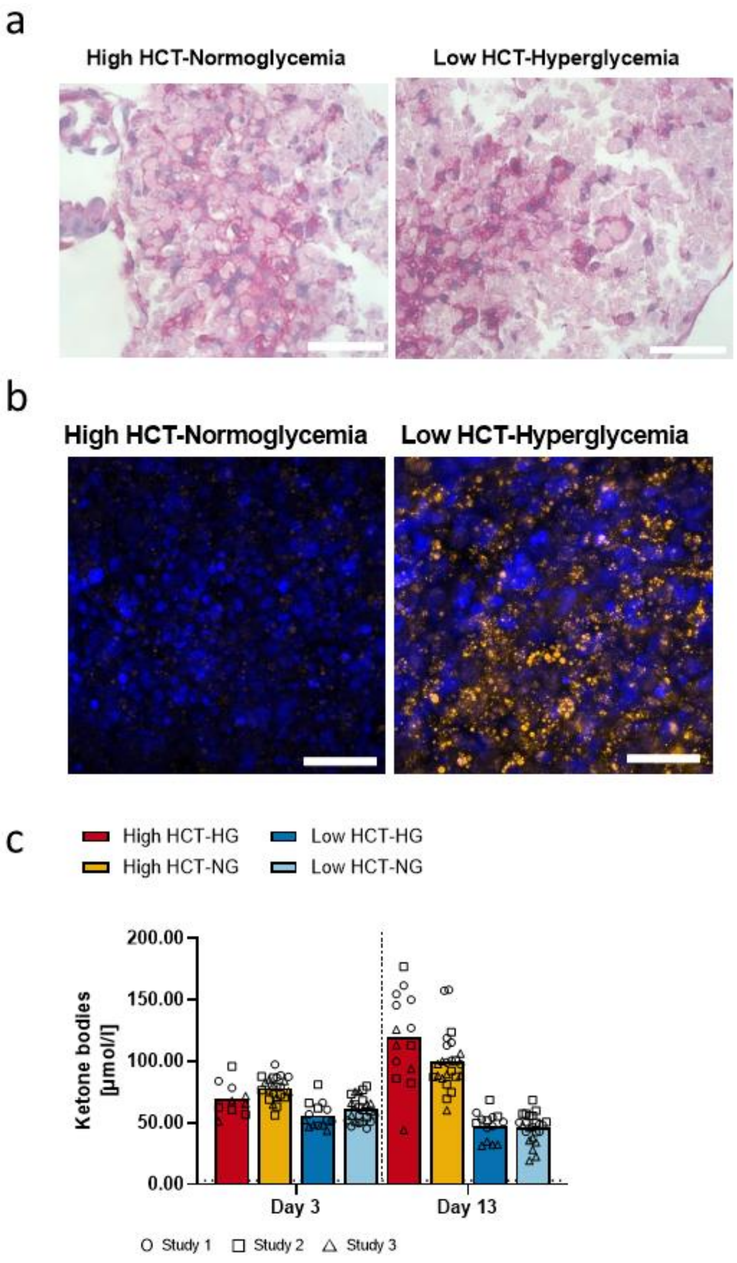

Characterization of HepaRG/HHSteC liver spheroids in normoglycemic high-HCT condition and in hyperglycemic low-HCT condition. (a) Glycogen storage visualized by periodic acid-Schiff (PAS) staining. Scale bar, 50 µm. (**b**) Intracellular lipid vesicles visualized by Nile Red staining (amber colour). Blue denotes DAPI-stained nuclei. Scale bar, 50 µm. (**c**) Ketone body synthesis represented by 3-hydroxybutyrate concentration in the co-culture supernatants. Bars show mean and symbols represent individual co-culture replicates from three independent studies (n values summarized in Table S1). Studies 1 and 2 were performed at TissUse and study 3 at AstraZeneca.

Fig. S7.

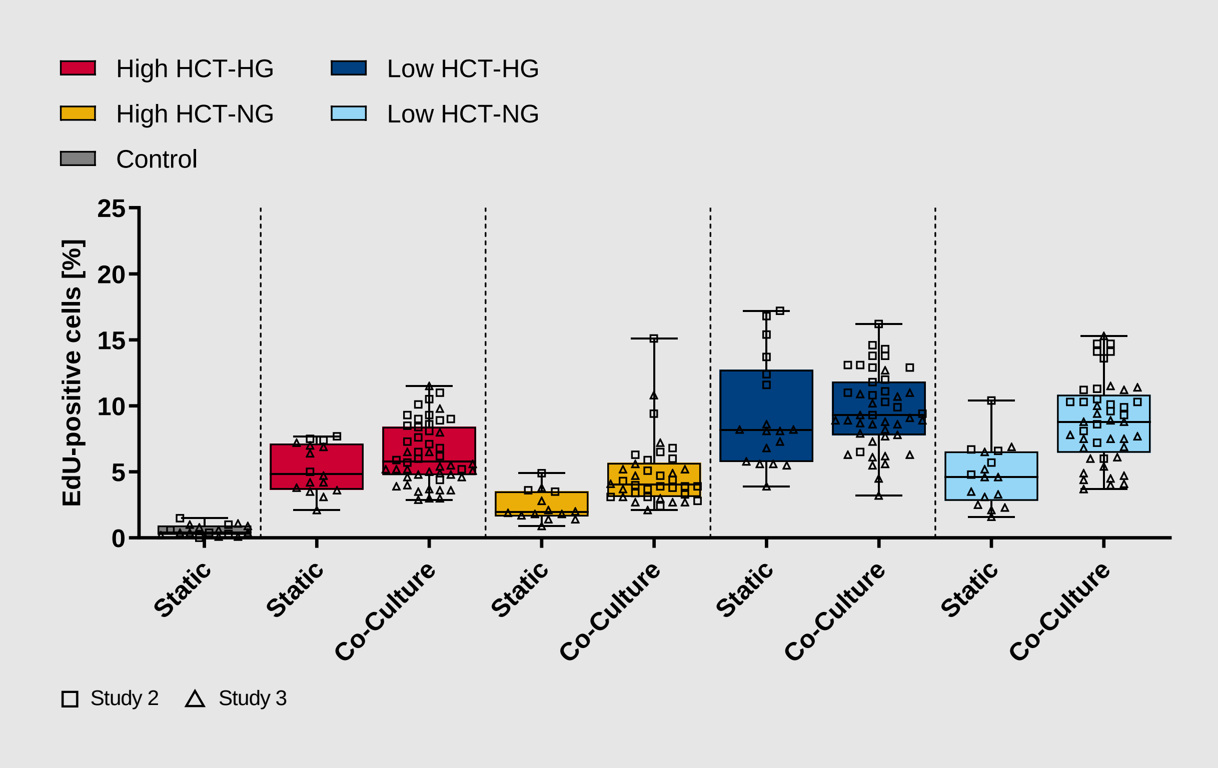

Islet proliferation in all studied medium conditions. Proliferation of islets in static monocultures and in chip co-culture with liver spheroids. In both culture systems, islets were maintained in the diseased (11 mM glucose, 50 µM HCT) and in the healthy (5.5 mM glucose, 10 nM HCT) condition and extracted for the proliferation assay at the end of the culture. Data shown as percentage of EdU-positive cells in a box-whisker plot with median and min-max values. Symbols represent individual islets from two independent co-culture studies (n values summarized in Table S1). Study 2 was performed at TissUse and study 3 at AstraZeneca.

Fig. S8.

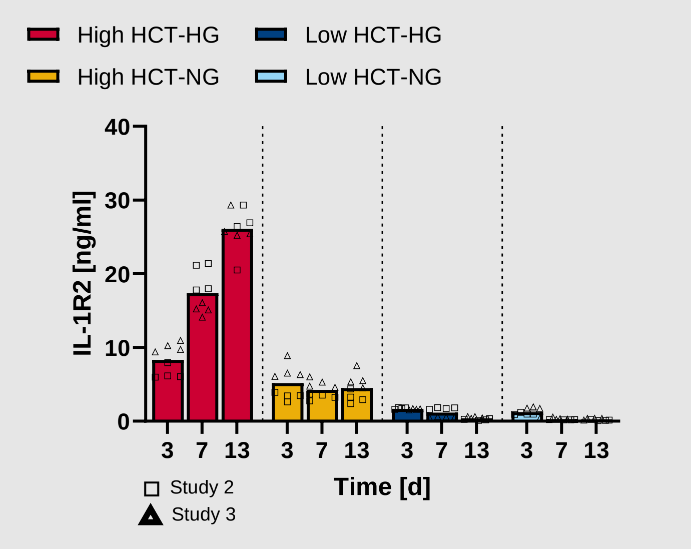

Secretion of IL-1R2 in all studied medium conditions. IL-1R2 concentration in the pancreas-liver co-cultures over time. Bars show mean and symbols represent co-culture replicates from study 2 and 3 (Study 1: n = 4, Study 2: n = 4). Study 2 was performed at TissUse and study 3 at AstraZeneca.

Fig. S9.

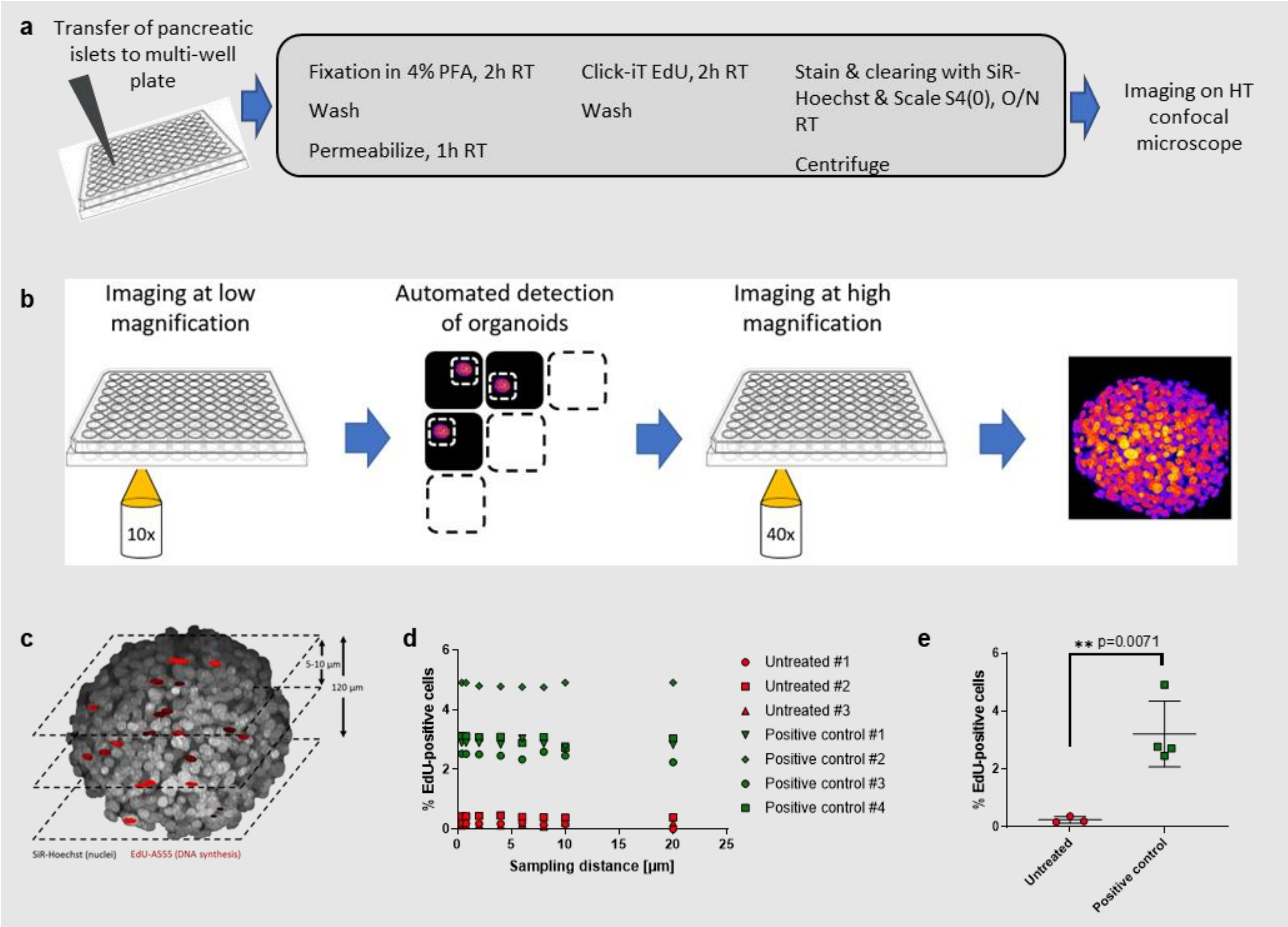

Detection of cell proliferation in human pancreatic islets using EdU incorporation, automated high-throughput confocal microscopy, and optical slicing. (**a**) Method description for pancreatic islet preparation, clearing, and staining for high-throughput imaging. (**b**) Schematic of automated detection of islets for high resolution imaging using Search First algorithm in multi-well plates. (**c**) Schematic of optical slicing of islets by confocal microscopy. (**d**) The percentage of EdU-positive cells is largely independent of the sampling distance (range 0.4–20 µm) as shown with untreated islets and islets treated with MST1 kinase inhibitor[71]. (**e**) The percentage of EdU-positive cells in untreated islets and in islets treated with MST1 kinase inhibitor. Data represents mean and SD. Significance determined by unpaired t-test.

Fig. S10.

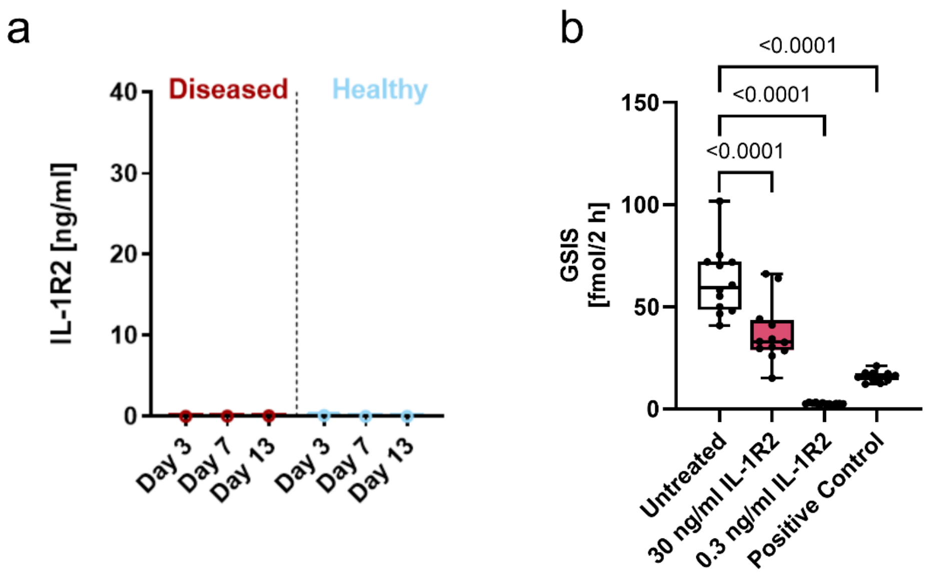

Secretion and effect of IL-1R2. (a) IL-1R2 concentration in static islet monocultures in the diseased (11 mM glucose, 50 µM HCT) and healthy (5.5 mM glucose, 10 nM HCT) conditions over time. Symbols represent individual islets (n = 1). (**b**) IL-1R2 treatment decreases glucose stimulated insulin secretion (GSIS) both at low (0.3 ng/mL) and high (30 ng/mL) IL-1R2 dose in islets monocultured in static condition in culture medium provided by the islet manufacturer. Data represents GSIS response after high-glucose stimulation (16.8 mM) in a box-whisker plot with median and min-max values. Control, 0 ng/mL IL-1R2. Positive control: hyperglycemic low-HCT co-culture medium. Symbols represent individual islets (n = 12 for untreated, 30 ng/mL IL-1R2 and 0.3 ng/mL IL-1R2, n = 11 for positive control). Differences to the untreated control were evaluated by one-way ANOVA using Dunnett’s multiple comparisons post-hoc test. Study was performed at AstraZeneca.

Fig. S11.

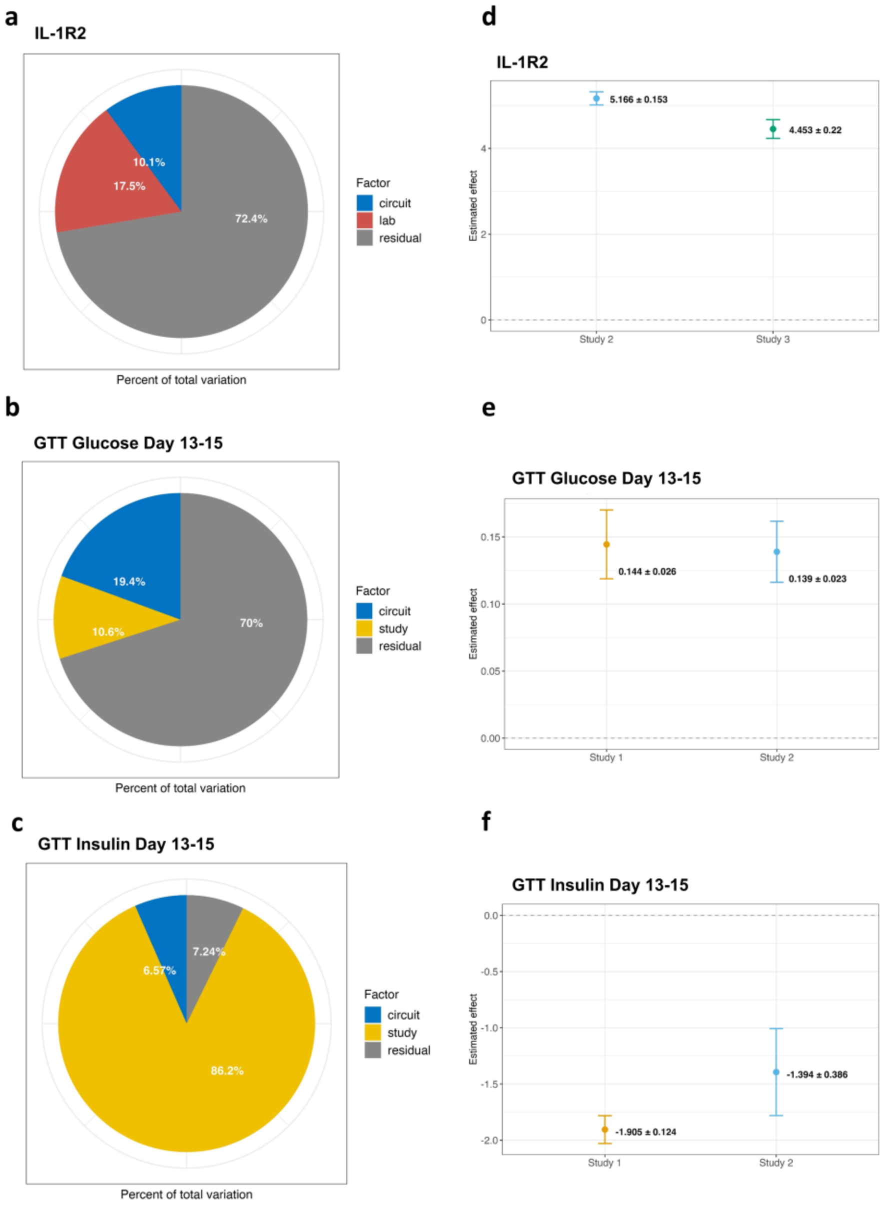

Evaluation of technical sources of variation and reproducibility. (a, b, c) The proportion (%) of total variance associated with nested factors lab (laboratory), study, circuit, and residuals, respectively for the readouts IL-1R2 **(a)**, GTT glucose at day 13-15 **(b)**, and GTT insulin at day 13-15 **(c)**. **(d, e, f)** The estimated difference between diseased and healthy conditions and the associated standard error for each study are shown for IL-1R2 **(d)**, GTT glucose **(e)**, and GTT insulin **(f)**. These estimates are obtained after mixed models have been fit to data for each study independently, and contrasts have been tested at day 13. For GTT readouts only data from days 13-15 have been included and contrasts are tested at day 15.

Fig. S12

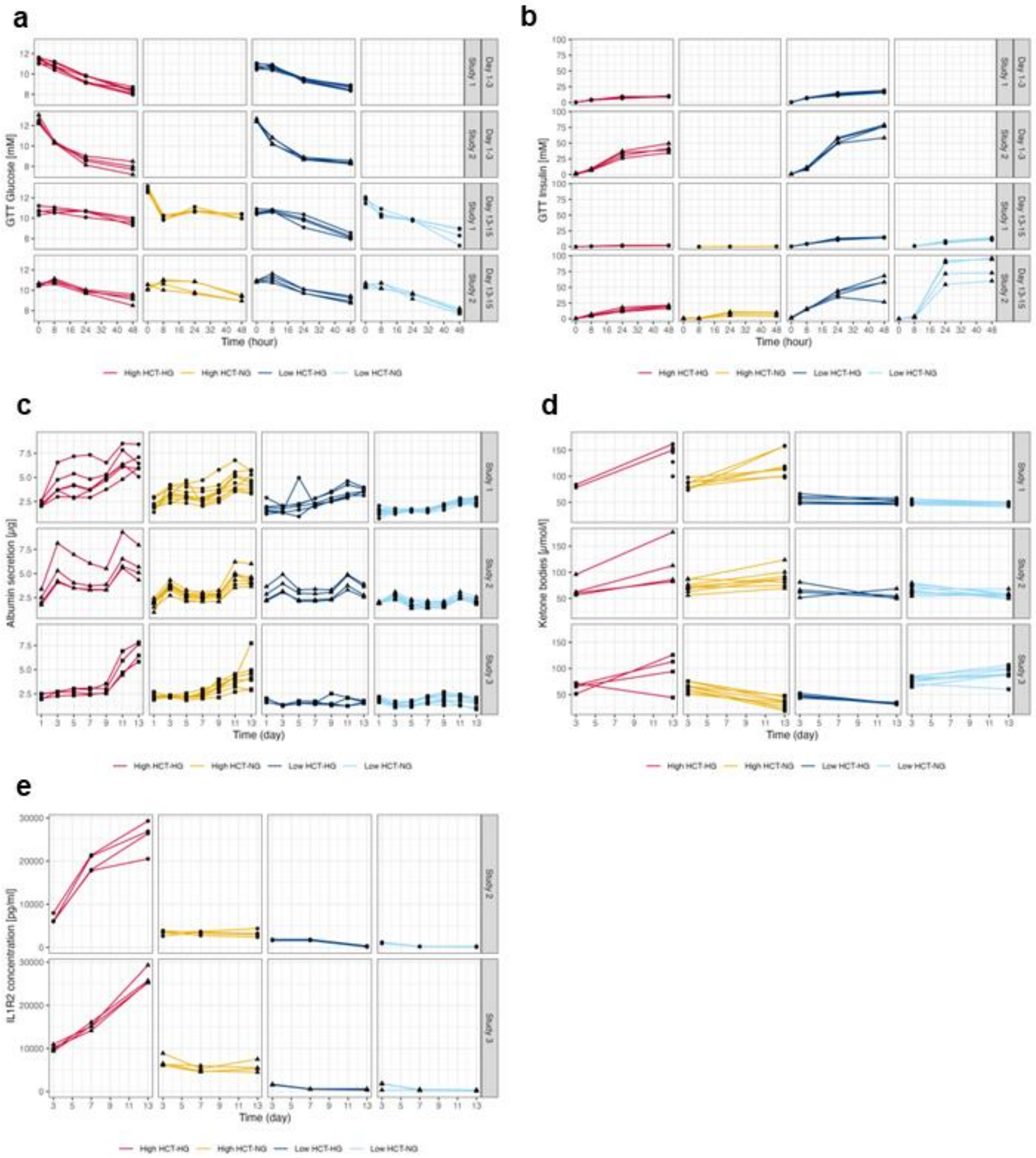

On-chip readout results from individual chip circuits. Results presented as line plots from individual circuits from all studies for GTT glucose **(a)**, GTT insulin **(b)**, albumin **(c)**, ketone bodies **(d)**, and IL-1R2 **(e)**. Studies 1 and 2 were performed at TissUse and study 3 at AstraZeneca.

Table S1. Experimental design of the pancreas-liver MPS. Numbers indicate sample size (n) for each readout.

Study	Lab	HepaRG lot	Islet Donor	Condition	No. of circuits	On-chip readouts				End-point readouts with extracted organ models				
						Insulin and Glucose GTT	Albumin	Ketone bodies	IL1-R2	qPCR	GSIS (Static/Co-Culture)	EdU (Static/Co-Culture)	RNA-Seq	Proteomics
**Hypothesis testing**	Astra Zeneca	Lot 1 (HPR116239-TA08)	Donor 1	High hydrocortisone-hyperglycemia	3	3 (Day 1-3)<br>3 (Day 7-9)<br>3 (Day 13-15)	-	-	-	-	-	-	-	-
				High hydrocortisone-normoglycemia	3	3 (Day 1-3)<br>3 (Day 7-9)<br>3 (Day 13-15)	-	-	-	-	-	-	-	-
**Study 1**	TissUse	Lot 2 (HPR116NS080003)	Donor 3	High hydrocortisone-hyperglycemia	6	6 (Day 1-3)<br>4 (Day 13-15)	6	6	-	4	5 / 11	-	-	-
				High hydrocortisone-normoglycemia	6	6 (Day 1-3)<br>4 (Day 13-15)	10	10	-	4	4 / 11	-	-	-
				Low hydrocortisone-hyperglycemia	10	4 (Day 13-15)	6	6	-	4	7 / 11	-	-	-
				Low hydrocortisone-normoglycemia	10	4 (Day 13-15)	10	8	-	4	10 / 11	-	-	-
**Study 2**	TissUse	Lot 3 (HNS1014)	Donor 4	High hydrocortisone-hyperglycemia	4	4 (Day 1-3)<br>4 (Day 13-15)	4	4	4	4	5 / 12	4 / 23	-	-
				High hydrocortisone-normoglycemia	4	4 (Day 1-3)<br>4 (Day 13-15)	8	8	4	4	5 / 11	3 / 23	-	-
				Low hydrocortisone-hyperglycemia	8	4 (Day 13-15)	4	4	4	4	6 / 12	6 / 19	-	-
				Low hydrocortisone-normoglycemia	8	4 (Day 13-15)	8	8	4	4	6 / 12	5 / 19	-	-
**Study 3**	Astra Zeneca	Lot 1 (HPR116239-TA08)	Donor 5	High hydrocortisone-hyperglycemia	4	-	4	4	4	-	8 / 8	12 / 27	-	-
				High hydrocortisone-normoglycemia	4	-	8	8	4	-	8 / 8	11 / 15	-	-
				Low hydrocortisone-hyperglycemia	8	-	4	4	4	-	8 / 8	12 / 27	-	-
				Low hydrocortisone-normoglycemia	8	-	8	8	4	-	8 / 8	12 / 26	-	-
**Combined omics study 1**	TissUse	Lot 2 (HPR116NS080003)	Donor 3	High hydrocortisone-hyperglycemia	2	-	-	-	-	-	-	-	2	2 circuits pooled
				High hydrocortisone-normoglycemia	2	-	-	-	-	-	-	-	2	2 circuits pooled
**Combined omics study 2**	TissUse	Lot 2 (HPR116NS080003)	Donor 6	High hydrocortisone-hyperglycemia	2	-	-	-	-	-	-	-	2	2 circuits pooled
				High hydrocortisone-normoglycemia	2	-	-	-	-	-	-	-	2	2 circuits pooled
**Combined omics study 3**	Astra Zeneca	Lot 4 (HPR116222-TA08)	Donor 7	High hydrocortisone-hyperglycemia	2	-	-	-	-	-	-	-	2	2 circuits pooled
				High hydrocortisone-normoglycemia	2	-	-	-	-	-	-	-	2	2 circuits pooled
**Proteomics study 4**	Astra Zeneca	Lot 4 (HPR116222-TA08)	Donor 8	High hydrocortisone-hyperglycemia	2	-	-	-	-	-	-	-	-	2 circuits pooled
				High hydrocortisone-normoglycemia	2	-	-	-	-	-	-	-	-	2 circuits pooled

Table S2.
Islet donors used in the pancreas-liver co-cultures.

Donor Nr.	Study	Sex	Age	BMI	HbA1c
Donor 1	Hypothesis testing	Male	52 years	29.6	5.4%
Donor 2	Hydrocortisone dose response	Female	57 years	28.97	5.6%
Donor 3	Study 1/ Combined omics study 1	Male	29 years	22.2	5.5%
Donor 4	Study 2	Male	26 years	24.1	5.1%
Donor 5	Study 3	Male	55 years	30.9	5.6%
Donor 6	Combined omics study 2	Female	57 years	21.35	5.8%
Donor 7	Combined omics study 3	Male	45 years	29.84	5.1%
Donor 8	Proteomics study 4	Male	55 years	26.81	5.8%
Donor 9	IL-1R2 treatment	Female	32 years	25.6	5.1%

Table S3.

Primers used in the qPCR.

Gene symbol	Name	Forward primer (5' - 3')	Reverse primer (5' - 3')
ACACA	Acetyl-CoA carboxylase alpha	AATAAGGATCTGGCGGAGTGG	GCTCGCTGAGTGGGTGATATG
ACACB	Acetyl-CoA carboxylase 2	GCCGACTTCCATGACACACC	TGTTCCAGCCACTGCACAAC
ACADM	Acyl-Coenzyme A dehydrogenase	TGTTTTAATTGGTGACGGAGCTG	ACCAGAATCAACCTCCCAAGC
ACOX1	Acyl-CoA oxidase 1	GCAAGGAGGTAGCTTGGAACC	GATGCTCCCCTGAAGGAAATC
ACTA2	Alpha-actin-2	AGAGACCCTGTTCCAGCCATC	CGTGATCTCCTTCTGCATTCG
AHSG	Alpha 2-HS glycoprotein	CGCAAAATGTGATTCCAGTCC	CCGTTGTTCTGAGCGTTGAAG
ALB	Albumin	TCAGCTCTGGAAGTCGATGAAAC	AGTTGCTCTTTTGTTGCCTTGG
APOB	Apolipoprotein B	AGGCATCTCCACCTCAGCAG	GCTGCCTCTTCTTCCCAATTAAC
BDH1	3-hydroxybutyrate dehydrogenase 1	AGCAGGTGGCAGAAGTGAACC	CTACCCCGAACTTGGTGATGC
BSEP/ABCB11	Bile salt export pump/ATP binding cassette subfamily B member 11	GCAGACACTGGCGTTTGTTG	ATGTTTGAGCGGAGGAACTGG
CPS1	Carbamoyl-phosphate synthase 1	CCCAGCCTCTCTTCCATCAG	GCGAGATTTCTGCACAGCTTC
CPT1A	Carnitine palmitoyltransferase 1A	CGTCACCTCTTCTGCCTTTACG	CTCCGCTGGACACGTACTCTG
CYP3A4	Cytochrome P450 family 3 subfamily A member 4	GGAAGTGGACCCAGAAACTGC	TTACGGTGCCATCCCTTGAC
DGAT1	Diacylglycerol O-Acyltransferase 1	GGCCTTACCTGGCTACACTGG	CATTGCCACTCCCATTCTTTG
FASN	Fatty acid synthase	ACGGACATGGAGCACAACAG	GGTACTTGGCCTTGGGTGTG
G6PC	Glucose-6-phosphatase catalytic subunit	GCTGAATGTCTGTCTGTCACGAA	CGAAGCTGAACAGGAAGAAGGT
GYS2	Glycogen synthase 2	GCCAGACACCTGACATTAAGCA	TGAGACCCTGAAGGAGAAGGTG
HADHA	Hydroxyacyl-CoA dehydrogenase trifunctional multienzyme complex subunit alpha	TGGTAGAAGCATTCGTGCAGAC	GGCATACGCTGTCAATTTTTCC
HMGCL	3-hydroxy-3-methylglutaryl-CoA lyase	TCTACTCAATGGGCTGCTACGAG	TCCTGCCACAGAAGAGTCCAC
HMGCS1	3-hydroxy-3-methylglutaryl-CoA synthase 1	TTGAGTCCAGCTCTTGGGATG	CACTGGGCATGGATCTTTTTG
HMGCS2	3-hydroxy-3-methylglutaryl-CoA synthase 2	CCACCACTCTGCCCAAGAAC	ACACACTTTCGGGAGGCTAGG
HNF4	Hepatocyte nuclear factor 4 alpha	ATACGCATCCTTGACGAGCTG	CTGGCGGTCGTTGATGTAGTC
IDE	Insulin degrading enzyme	TGGATTCTTGTCCTGTTGTTGG	TCAGAGTTTTGCAGCCATGAAG
INSR	Insulin receptor	CACGCCAAAGGACCACATGTC	CGAGAAACTGCATGGTCGCC
MLXIPL	MLX interacting protein like	AGCTGCGGGATGAGATTGAG	TCAAACAGAGGCCGGATGAG
MRP2/ABCC2	Multidrug resistance-associated protein 2/ATP binding cassette subfamily C member 2	GCATCCACAGACATCAGGTTCAC	CTGCGGCTCTCATTCAGTCTTTC
MTTP	Microsomal Triglyceride Transfer Protein	TGCAATGGAGTTTAGCTTGTGG	GTTTCCAGGCCAGCTTTCAC
PCK1	Phosphoenolpyruvate carboxykinase 1	CATGAGATCAGAGGCCACAGC	AATTTGCCTTCCTTGTCCTTCC
PLIN2	Perilipin 2	TTCACTCCCGTGCCTACCAG	CATCATATCCAATGCTCCTTTTCC
PYGL	Glycogen phosphorylase L	GATGTGGCTGCTTTGGACAAG	TCTTGACACTTGACATAGGCTTCG
SCD	Stearoyl-CoA desaturase	GCCACCGCTCTTACAAAGCTC	CGTCGGGAATTATGAGGATCAG
SLC2A2	Solute carrier family 2 member 2/GLUT2	TGGTTCATGGTGGCTGAGTTT	AGGGTAAAGGCCAGGAGCAC
SREBF1	Sterol regulatory element binding transcription factor 1	TCTGAGAGACCCCTGCCCAG	CTGCACGGCCTTGTCAATGG
SREBF2	Sterol Regulatory Element Binding Transcription Factor 2	GCTGTGCGCTCTCATTTTACC	GACGTTGAGGCTGCTCCATAG
TBP	TATA-box binding protein	CCTTGTGCTCACCCACCAAC	TCGTCTTCCTGAATCCCTTTAGAATAG
