# Supplementary material for: Normoglycemia and physiological cortisone level maintain glucose homeostasis in a pancreas-liver microphysiological system
Source: Commun Biol. 2024 Jul 18;7:877. doi: 10.1038/s42003-024-06514-w (PMC11258270; doi:10.1038/s42003-024-06514-w)
Supplement: Supplementary file 5 — Reporting summary [file 42003_2024_6514_MOESM5_ESM.pdf]

nature portfolio

Corresponding author(s): Liisa Vilén

Last updated by author(s): Jun 8, 2024

Reporting Summary

Nature Portfolio wishes to improve the reproducibility of the work that we publish. This form provides structure for consistency and transparency in reporting. For further information on Nature Portfolio policies, see our Editorial Policies and the Editorial Policy Checklist.

Statistics

For all statistical analyses, confirm that the following items are present in the figure legend, table legend, main text, or Methods section.

n/a	Confirmed	
☐	☒	The exact sample size (*n*) for each experimental group/condition, given as a discrete number and unit of measurement
☐	☒	A statement on whether measurements were taken from distinct samples or whether the same sample was measured repeatedly
☐	☒	The statistical test(s) used AND whether they are one- or two-sided<br>*Only common tests should be described solely by name; describe more complex techniques in the Methods section.*
☐	☒	A description of all covariates tested
☐	☒	A description of any assumptions or corrections, such as tests of normality and adjustment for multiple comparisons
☐	☒	A full description of the statistical parameters including central tendency (e.g. means) or other basic estimates (e.g. regression coefficient) AND variation (e.g. standard deviation) or associated estimates of uncertainty (e.g. confidence intervals)
☐	☒	For null hypothesis testing, the test statistic (e.g. *F*, *t*, *r*) with confidence intervals, effect sizes, degrees of freedom and *P* value noted<br>*Give P values as exact values whenever suitable.*
☒	☐	For Bayesian analysis, information on the choice of priors and Markov chain Monte Carlo settings
☐	☒	For hierarchical and complex designs, identification of the appropriate level for tests and full reporting of outcomes
☐	☒	Estimates of effect sizes (e.g. Cohen's *d*, Pearson's *r*), indicating how they were calculated

Our web collection on statistics for biologists contains articles on many of the points above.

Software and code

Policy information about availability of computer code

Data collection

- Supernatants of cell culture medium were analysed for albumin and ketone bodies on an Indiko Plus chemical analyzer (Thermo Fisher Scientific) using the Indiko software (version 7.0.2).
- QuantStudio Design & Analysis software was used to analyse qPCR data generated usin the QuantStudio 5 Real-Time PCR System.
- Wako Software Suite was used to acquire images on a CellVoyager 7000 high-throughput spinning disc confocal microscope.
- Analysis of total number of EdU-positive nuclei was performed using Columbus™ Image Data Storage and Analysis system (ver. 2.8.1, Perkin Elmer).
- GraphPad Prism software (Version 8) was used to plot the data and perform comparative analysis between the means of different conditions.
- Computations for mathematical modelling were carried out in MATLAB R2022b (The Mathworks Inc., Natick, Massachusetts, USA) using IQM tools (IntiQuan GmbH, Basel, Switzerland) and the MATLAB Global Optimization toolbox, as well as in Python (v 3.9.13).
- RNASeq data was analysed using bcbio (version 1.1.0) and differential analysis was performed with DESeq2 (version 1.18.1).
- Proteomic data files were merged for identification and relative quantification using Proteome Discoverer version 2.1.1.21 (Thermo Fisher Scientific).
- Swiss-Prot Human database was used for the database search, using the Mascot search engine v. 2.5.1 (Matrix Science, London, UK).
- Proteomic data was compared to RNASeq results by pairing log2 fold-changes at the gene level and plotted in Fig. 6B. Data was plotted with R version 4.0.2 with ggplot2 version 3.3.5.
- Evaluation of technical confounders and reproducibility was conducted using the R Statistical language (version 4.2.1).
- Mixed modelling was performed using the lmer() function from the lme4 package (version 1.1.34), with the Nelder-Mead optimiser to address convergence issues. Significance was tested through summary functions in the lmerTest package (version 3.1.3), using Satterthwaite's correction for degrees of freedom. Contrasts for estimated marginal means were computed using the emmeans package (version 1.8.2).
- ICC procedure used was ICC2 from the R package psych (version 2.4.1).

Data analysis

The code to reproduce the results in this manuscript related to mathematical modeling is publicly available at: https://github.com/belencasasgarcia/Insulin-resistance. The code for the mixed model statistical analysis is publicly available at https://doi.org/10.5281/zenodo.11545665

For manuscripts utilizing custom algorithms or software that are central to the research but not yet described in published literature, software must be made available to editors and reviewers. We strongly encourage code deposition in a community repository (e.g. GitHub). See the Nature Portfolio guidelines for submitting code & software for further information.

Data

Policy information about availability of data

All manuscripts must include a data availability statement. This statement should provide the following information, where applicable:

- Accession codes, unique identifiers, or web links for publicly available datasets
- A description of any restrictions on data availability
- For clinical datasets or third party data, please ensure that the statement adheres to our policy

The code for data analysis, visualization and mathematical modelling is publicly available on GitHub. The code for the mixed model statistical analysis is publicly available on Zenodo. Transciptomics data is publicly available on GEO. Proteomics data is publicly available on PRIDE. Links are provided in the manuscript, Data and materials availability section.

Human research participants

Policy information about studies involving human research participants and Sex and Gender in Research.

Reporting on sex and gender	*Use the terms sex (biological attribute) and gender (shaped by social and cultural circumstances) carefully in order to avoid confusing both terms. Indicate if findings apply to only one sex or gender; describe whether sex and gender were considered in study design whether sex and/or gender was determined based on self-reporting or assigned and methods used. Provide in the source data disaggregated sex and gender data where this information has been collected, and consent has been obtained for sharing of individual-level data; provide overall numbers in this Reporting Summary. Please state if this information has not been collected. Report sex- and gender-based analyses where performed, justify reasons for lack of sex- and gender-based analysis.*
Population characteristics	*Describe the covariate-relevant population characteristics of the human research participants (e.g. age, genotypic information, past and current diagnosis and treatment categories). If you filled out the behavioural & social sciences study design questions and have nothing to add here, write "See above."*
Recruitment	*Describe how participants were recruited. Outline any potential self-selection bias or other biases that may be present and how these are likely to impact results.*
Ethics oversight	*Identify the organization(s) that approved the study protocol.*

Note that full information on the approval of the study protocol must also be provided in the manuscript.

Field-specific reporting

Please select the one below that is the best fit for your research. If you are not sure, read the appropriate sections before making your selection.

☒ Life sciences ☐ Behavioural & social sciences ☐ Ecological, evolutionary & environmental sciences

For a reference copy of the document with all sections, see nature.com/documents/nr-reporting-summary-flat.pdf

Life sciences study design

All studies must disclose on these points even when the disclosure is negative.

Sample size	No statistical methods were used to predetermine the sample size. Sample sizes were determined based on previous published literature. Studies were done in two independent laboratories.
Data exclusions	No data was excluded.
Replication	Studies were replicated in two separate laboratories, total three independent studies were performed each having 3-4 chips per treatment group. All attempts were successful.
Randomization	Liver spheroids and pancreatic islets were randomly chosen for different treatment groups.
Blinding	Blinding was not done.

Reporting for specific materials, systems and methods

We require information from authors about some types of materials, experimental systems and methods used in many studies. Here, indicate whether each material, system or method listed is relevant to your study. If you are not sure if a list item applies to your research, read the appropriate section before selecting a response.

Materials & experimental systems

n/a	Involved in the study
☒	☐ Antibodies
☐	☒ Eukaryotic cell lines
☒	☐ Palaeontology and archaeology
☒	☐ Animals and other organisms
☒	☐ Clinical data
☒	☐ Dual use research of concern

Methods

n/a	Involved in the study
☒	☐ ChIP-seq
☒	☐ Flow cytometry
☒	☐ MRI-based neuroimaging

Eukaryotic cell lines

Policy information about cell lines and Sex and Gender in Research

Cell line source(s)	Differentiated HepaRG hepatocyte-like cells (HPR116080, Biopredic, Lot HPR116NS080003 and HPR116239-TA08 or NSHPRG, Lonza, Lot HNS1014
Authentication	Differentiated HepaRG cells were used in the study immediately after thawing, no further culture or passaging. Thus, authentication process was not done.
Mycoplasma contamination	Cells tested negative for mycoplasma contamination.
Commonly misidentified lines (See ICLAC register)	Not applicable.
